# Effects of metformin and statins on outcomes in men with castration-resistant metastatic prostate cancer: Secondary analysis of COU-AA-301 and COU-AA-302

**DOI:** 10.1016/j.ejca.2022.03.042

**Published:** 2022-05-11

**Authors:** Brooke E. Wilson, Andrew J. Armstrong, Johann de Bono, Cora N. Sternberg, Charles J. Ryan, Howard I. Scher, Matthew R. Smith, Dana Rathkopf, Christopher J. Logothetis, Kim N. Chi, Robert J. Jones, Fred Saad, Peter De Porre, NamPhuong Tran, Peter Hu, Silke Gillessen, Joan Carles, Karim Fizazi, Anthony M. Joshua

**Affiliations:** a Princess Margaret Cancer Centre, Toronto, Canada; b Faculty of Medicine, University of New South Wales, Kensington, Australia; c Kinghorn Cancer Centre, St Vincents Hospital, Darlinghurst, Sydney, Australia; d Duke Cancer Institute Center for Prostate and Urologic Cancers, Durham NC, USA; e The Institute of Cancer Research and Royal Marsden Hospital, London, UK; f Englander Institute for Precision Medicine, Weill Cornell Medicine, New York-Presbyterian, New York, NY 10021, USA; g Division of Hematology, Oncology and Transplantation, University of Minnesota, Minneapolis, MN, USA; h Department of Medicine, Memorial Sloan-Kettering Cancer Center, New York, NY, USA; i Massachusetts General Hospital Cancer Center, 55 Fruit Street, Boston, MA, 02114, USA; j Genitourinary Oncology Service, Department of Medicine, Memorial Sloan Kettering Cancer Center, New York, NY USA; k Genitourinary Medical Oncology, The University of Texas MD Anderson Cancer Center, Houston, TX, USA; l BC Cancer, Vancouver, Canada; m Institute of Cancer Sciences, University of Glasgow, Beatson West of Scotland Cancer Centre, Glasgow, UK; n Department of Urology, University of Montreal Hospital Center, Montreal, Quebec, Canada; o Janssen R&D, Beerse, Belgium; p Janssen R&D, Los Angeles, CA, USA; q Janssen LLC. Raritan, New Jersey, USA; r Institute of Oncology of Southern Switzerland, Bellinzona, Switzerland and Universita Della Svizzera Italiana, Lugano, Switzerland and University of Manchester, UK; s Vall D’Hebron Institute of Oncology, Vall D’Hebron University Hospital, Barcelona, Spain; t Institut Gustave Roussy, University of Paris Saclay, Villejuif, France

**Keywords:** Metastatic castration-resistant prostate cancer, Metformin, Statins, Abiraterone acetate

## Abstract

**Background:**

The associations of metformin and statins with overall survival (OS) and prostate specific antigen response rate (PSA-RR) in trials in metastatic castration-resistant prostate cancer remain unclear.

**Objective:**

To determine whether metformin or statins ± abiraterone acetate plus prednisone/prednisolone (AAP) influence OS and PSA-RR.

**Design, setting and participant:**

COU-AA-301 and COU-AA-302 patients were stratified by metformin and statin use. Cox proportional hazards models were used to estimate hazards ratio (HR) stratified by concomitant medications, and a random effects model was used to pool HR. We compared PSA-RR using Chi χ^2^ test.

**Results:**

In COU-AA-301-AAP, metformin was associated with improved PSA-RR (41.1% versus 28.6%) but not prolonged OS. In COU-AA-301-placebo-P, there was no association between metformin and prolonged OS or PSA-RR. In COU-AA-302-AAP, metformin was associated with prolonged OS (adjHR 0.69, 95% CI 0.48–0.98) and improved PSA-RR (72.7% versus 60.0%). In COU-AA-302-P, metformin was associated with prolonged OS (adjHR 0.66, 95% CI 0.47–0.93). In pooled analysis, OS was prolonged among those treated with metformin (pooled HR 0.77, 95% CI 0.62–0.95).In COU-AA-301-AAP, statins were associated with an improved OS (adjHR 0.76, 95% CI 0.62–0.93), while there was no difference in COU-AA-301-P. There was no association with statins and OS in either COU-AA-302 groups. When pooling HR, OS was prolonged among those treated with statins (pooled HR 0.78, 95% CI 0.68–0.88).

**Conclusion:**

Within the limitations of post-hoc sub-analyses, metformin and statins are associated with a prolonged OS and increased PSA-RR, particularly in combination with AAP.

## 1. Introduction

The COU-AA-302 and COU-AA-301 studies demonstrated improved overall survival (OS) in men with metastatic castration-resistant prostate cancer (mCRPC) treated with abiraterone acetate plus prednisone/prednisolone (AAP) both before and after docetaxel chemotherapy, respectively, establishing new treatment paradigms [1, 2]. Despite this advance, the long-term prognosis of these patients remains limited and additional treatments to prolong survival are still needed.

Over the last 15 years, there has been increasing interest in the potential anti-neoplastic effect of commonly prescribed drugs most notably metformin, for which a reduced cancer incidence was first noted in population data in 2005 [3]. That was followed by several epidemiological studies showing a reduction in prostate cancer incidence and improved OS among patients receiving metformin [4-7]. In the largest meta-analysis to date that included 30 cohorts and over 1.6 million patients, patients with prostate cancer receiving metformin had improved OS compared to those who did not [8], a finding that remains controversial and may be subject to bias [9]. How the drug affects tumour growth is unclear but possibilities include activating AMPK that leads to inhibition of mTOR signalling, reduced fatty acid synthesis, and induction of cyclin-dependent kinase induced autophagy and apoptosis [10].

Similarly, adjunctive favourable effects of statins on prostate cancer progression have been reported. In one cohort of 14,000 men with the disease, statin use prior to diagnosis was associated with lower rates of prostate cancer-related deaths [11]. Cholesterol is a precursor to androgen synthesis, and the reduced availability of cholesterol may in turn reduce androgen production and the secretion of pro-inflammatory cytokines [12], slowing disease progression.

The objective of this study is to examine whether metformin and statin use in men being receiving AAP for mCRPC in the COU-AA-301 and COU-AA-302 trials improved OS and prostate specific antigen response rates (PSA-RR).

## 2. Methods

We performed a post-hoc secondary analysis of data collected in the COU-AA-301 and COU-AA-302 trials. In COU-AA-301, patients treated with post-docetaxel were randomised 2:1 to either AA (1000 mg) daily plus 5 mg bd prednisone or 5 mg bd prednisone alone. COU-AA-302 randomised chemotherapy-naïve patients in a 1:1 fashion to either AA (1000 mg) daily plus 5 mg bd of prednisone or 5 mg bd of prednisone alone. Detailed methods for these studies have been published previously [1,2]. We retrospectively extracted data on concomitant use of metformin and statins, recorded at study entry and examined for associations with (1) OS and (2) PSA-RR.

As per the study protocols, PSA-RR was defined as the proportion of patients achieving a decrease in PSA of at least 50% from the baseline PSA value confirmed at least 4 weeks or more after the initiation of treatment. Baseline variables were summarised by the presence or absence of concomitant medication. We examined for any differences between groups using the χ^2^ for categorical variables and student’s t test for continuous variables. Adverse event (AE) data for COU-AA-301 and 302, summarised by the use of concomitant medications and by treatment arm were tabulated, however, no statistical testing was performed due to the risk of multiple testing. OS was estimated using Kaplan-Meier methods and compared using the log rank test. Cox proportional hazards models were used to estimate the hazards ratio (HR) for OS by each concomitant medication. Variables chosen for inclusion in multivariate modelling were based on prior prognostic models predicting PFS or OS in patients with mCRPC treated with AAP either before [13] or after docetaxel [14]. Differences in the proportion of those with a PSA response were compared using χ^2^. A random effects model was used to pool unadjusted estimates of effect size for OS. Throughout the study, a two-tailed p < 0.05 was considered statistically significant for further hypothesis generation.

## 3. Results

Among the 1195 patients enrolled in COU-AA-301, 104 were reported to be receiving metformin (73 (9.2%) in the AAP group and 31 (7.8%) in the placebo group), and 339 (28.4%) to be receiving statins (236 (29.6%) in the AAP group and 103 (25.9%) in the placebo group). Among the 1088 patients enrolled in COU-AA-302, 134 were reported to be receiving metformin (66 (12.1%) in the AAP group and 68 (12.5%) in the placebo group), and 436 (40.1%) to be receiving statins (229 (41.9%) in the AAP group and 207 (38.2%) in the placebo group). Few patients in each group received combination metformin and statin, limiting any further analysis (COU-301-AAP n = 45; COU-301-placebo n = 15; COU-302-AAP n = 49; COU-302-placebo n = 48). Baseline characteristics stratified by statin/metformin use are presented in Supplemental Tables 1 and 2. Generally, patients taking statins or metformin had a higher rate of pre-existing cardiovascular disease and a higher body mass index.

### 3.1. The effect of metformin/statins on toxicity rates in each arm of COU-AA-301 and COU-AA-302

Total number of AE and grade 3 or 4 AE are presented in Supplemental Tables 3-6 and are broadly similar between all groups. Although absolute numbers were extremely low, there was a higher percentage of G3/4 cardiac disorder and G3/4 hypokalaemia in patients taking statins with AAP than those that did not take statins with AAP (COU-AA-301 – Cardiac disorder 8.5% (takers) versus 3.8% (non-takers), hypokalaemia 6.8% (takers) versus 4.3% (non-takers); COU-AA-302 – Cardiac disorder 8.7% (takers) versus 3.2% (non-takers), hypokalaemia 3.9% (takers) versus 1.6% (non-takers).

### 3.2. The effect of metformin on clinical outcomes

**Fig. 1.**
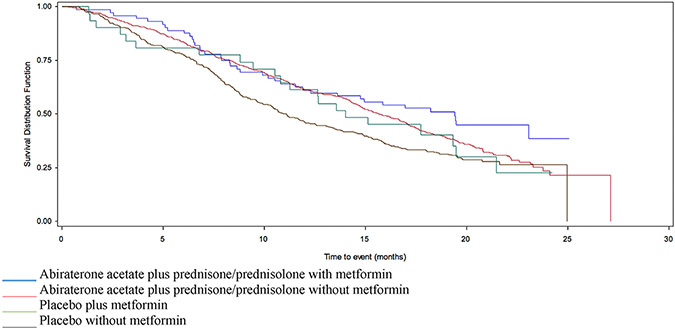
Survival in patients enrolled in COU-AA-301, stratified by AAP/placebo and metformin use. AAP, acetate plus prednisone/prednisolone.

In COU-AA-301-AAP, there was no definitive association with the median OS in those prescribed metformin (19.4 versus 15.6 months, HR 0.76 95% CI 0.55–1.05) (Table 1 and Fig. 1). However, the trend remained in multivariate analysis after adjusting for liver metastases, ECOG score, albumin, LDH and alkaline phosphatase levels (adjHR 0.71, 95% CI 0.5–1.006) (Table 2). The proportion of patients with PSA-RR was greater among the metformin takers than non-takers in the AAP arm (41.1% versus 28.6%) (Table 3). In COU-AA-301-placebo, metformin was not associated with a prolonged OS in univariate (14.0 versus 11.1 months, HR 0.85 95% CI 0.54–1.32) (Table 1 and Fig. 1) or multivariate analysis (Table 2). There was no difference in PSA-RR by metformin use status (3.2% versus 5.8%) (Table 3).

**Fig. 2.**
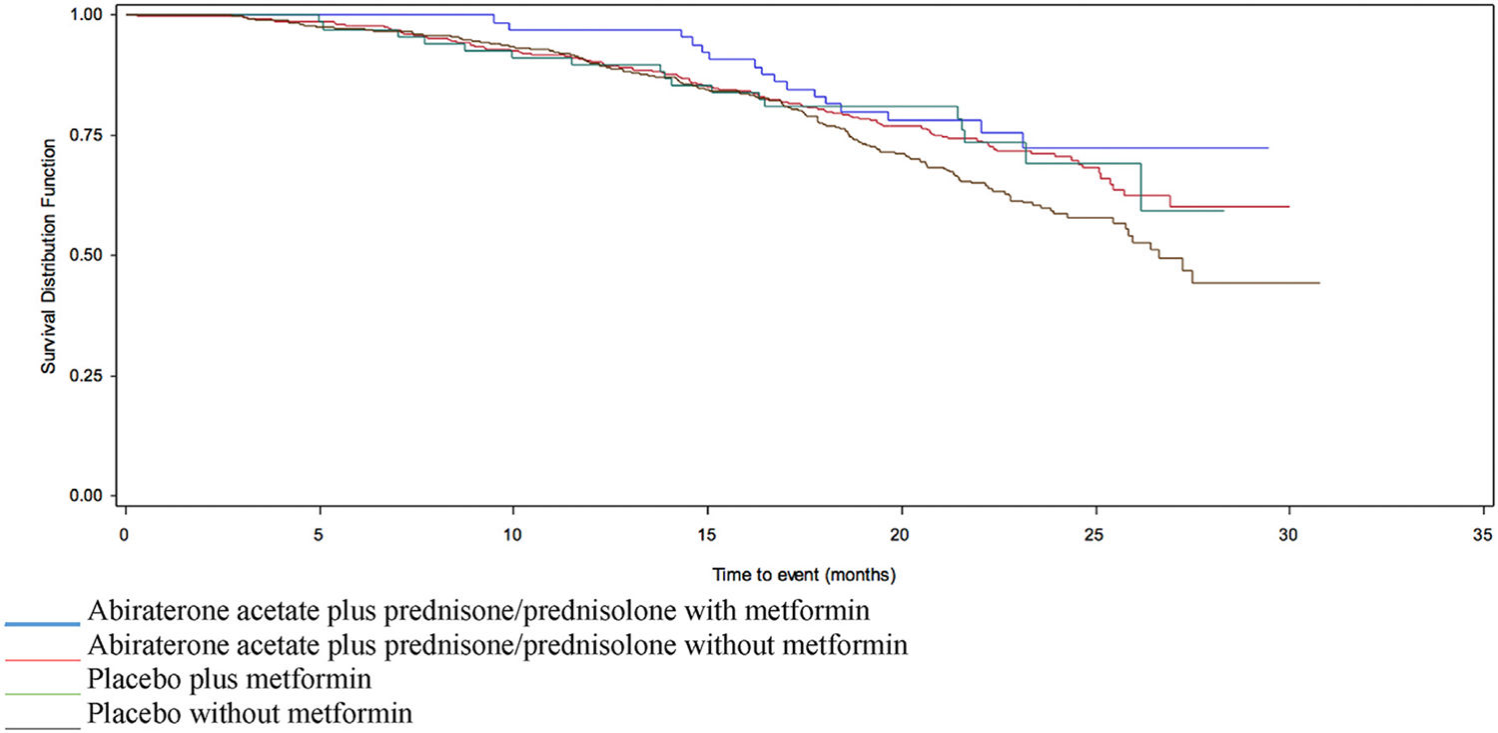
Survival in patients enrolled in COU-AA-302, stratified by AAP/placebo and metformin use. AAP, acetate plus prednisone/prednisolone.

In COU-AA-302-AAP, there was no association with OS by metformin use on univariate analysis (HR 0.81 95% CI 0.48–1.36) (Table 1 and Fig. 2). After adjusting for baseline factors including Brief Pain Inventory (BPI), age, LDH, alkaline phosphatase, bone metastases and baseline PSA, metformin was associated with prolonged OS (adjHR 0.69 95% CI 0.4–0.98) (Table 2). The proportion of patients with PSA-RR was also greater in those prescribed metformin (72.7% versus 60.0%) (Table 2). In COU-AA-302-placebo, there were no significant difference in PSA-RR (27.9% versus 23.3%) or OS by metformin use (HR 0.68 95% CI 0.42–1.11) (Tables 1 and 3 and Fig. 2). However, in multivariate analysis, metformin was associated with prolonged OS (adjHR 0.66 95% CI 0.47–0.93) (Table 2).

In summary, the use of metformin in COU-AA-301 was not associated with significant improvement in OS (albeit a trend) but there was an increased PSA-RR in those randomised to AAP, but not in those randomised to placebo. The use of metformin in COU-AA-302 was associated with improved OS in multivariate analysis and increased PSA-RR in those randomised to AAP. In COU-AA-302-placebo, there was no difference in PSA-RR, but an association with metformin use and a prolonged OS was seen. In pooling HR across both studies and treatment arms, OS was prolonged among those treated with metformin (HR 0.77, 95% CI 0.62–0.95) (Supplemental Figure 1).

### 3.3. The effect of statins on clinical outcomes

**Fig. 3.**
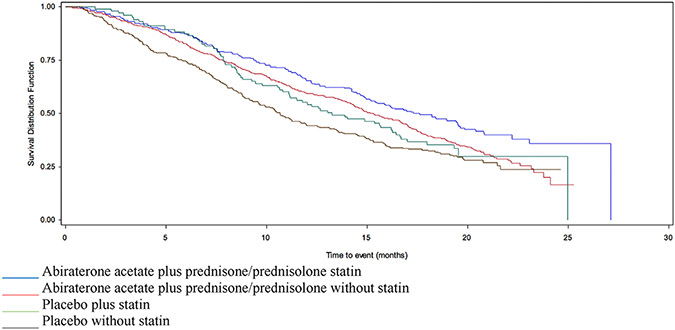
Survival in patients enrolled in COU-AA-301, stratified by AAP/placebo and statin use. AAP, acetate plus prednisone/prednisolone.

In COU-AA-301-AAP, concurrent statin use was associated with a longer median OS (17.6 versus 15.3 months, HR 0.76 95% CI 0.63–0.93) (Table 1 and Fig. 3). This association remained significant after adjusting for liver metastases, ECOG score, albumin, LDH, alkaline phosphatase and time from LHRH use to relapse <36 months (adjHR 0.76 95% CI 0.62–0.93) (Table 2). There was a higher PSA-RR among statin users (33.9% versus 28.0%) (Table 3). In COU-AA-301-placebo, the median OS was similar between statin user and non-user (13.2 versus 10.7 months) in both the univariate (HR 0.81 95% CI 0.62–1.07) and adjusted models (adjHR 0.96 95% CI 0.72–1.27) (Tables 1 and 2 and Fig. 3). There was no difference in PSA-RR by statins use (4.8% versus 5.8%) (Table 3).

**Fig. 4.**
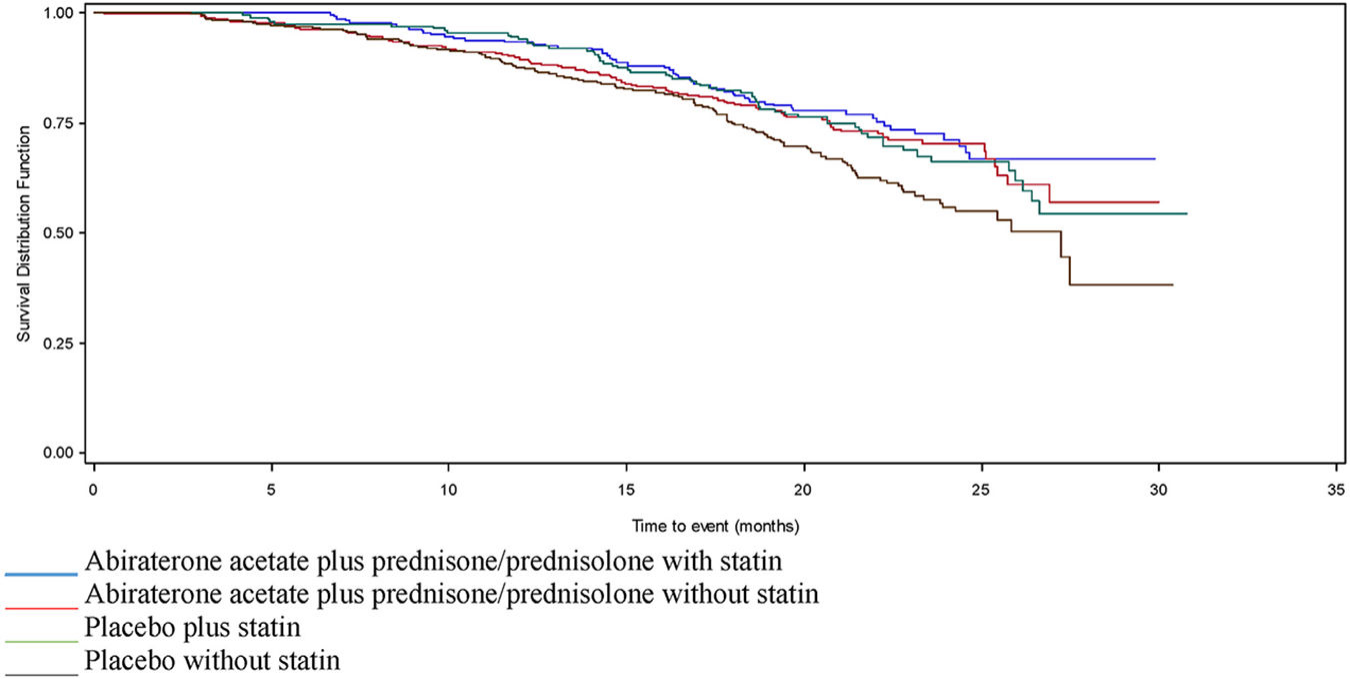
Survival in patients enrolled in COU-AA-302, stratified by AAP/placebo and statin use. AAP, acetate plus prednisone/prednisolone.

In COU-AA-302-AAP, there was no difference in OS in patients prescribed statins in either univariate or multivariate models (adjHR 1.00 95% CI 0.8–1.2), nor was there a difference in PSA-RR (60.7% versus 62.1%) (Tables 1-3 and Fig. 4). In COU-AA-302-placebo, median OS was longer among those prescribed statins in univariate analysis (HR 0.70 95% CI 0.52–0.96) (Table 1 and Fig. 4) but not in adjusted analysis (adjHR 0.88 95% CI 0.71–1.08) (Table 2). There was no difference in PSA-RR (23.3% versus 24.3%) (Table 3).

In summary, in COU-AA-301 statin use was associated with an improved OS and an increased PSA-RR in those randomised to AAP but not placebo. In COU-AA-302, there was no difference in OS or PSA response in the AAP or placebo groups between statin users and non-users. When pooling HR across all study and treatment arms, OS was prolonged among those treated with statins (pooled HR 0.78, 95% CI 0.68–0.88) (Supplemental Figure 2).

## 4. Discussion

The addition of metformin or statins to standard of care AAP is an attractive option to improve outcomes in men with prostate cancer due to the favourable safety profile, limited interaction with other drugs, low cost and widespread availability. Whether these medications improve patient outcomes when added to standard therapies remain controversial due to the lack of prospectively designed trials that specifically address the question and clear confounding effects. In this present analysis, we find that patients taking metformin in combination with AAP at study entry had an improved PSA-RR in both the chemotherapy-naïve (COU AA-302) and post-docetaxel (COU AA-301) treated patients. OS was prolonged in patients who are chemotherapy-naïve (COU AA-302) after adjusting for potential confounding baseline characteristics but showed only a trend towards improved OS in post-docetaxel (COU AA-301) treated patients. Statin use in combination with AAP was also associated with an improved OS in patients previously treated with docetaxel but had no effect in patients who are chemotherapy-naïve, after adjusting for baseline variables.

To date, there has been only one reported randomised trial examining the use of metformin in mCRPC, the phase II TAXOMET study in which 99 patients with mCRPC were randomised to treatment with docetaxel plus metformin versus docetaxel alone in order to increase the estimated PSA-RR from 45 to 60%. No differences in the primary end-point of PSA response rate was seen [15] and the median OS was 24.2 months (95% CI 17.2–33.7) in the combination arm versus 19.7 months (95% CI 14.8–36.8) with docetaxel alone, which did not meet statistical significance [15]. A small phase II pilot study of 25 men with mCRPC demonstrated that the addition of metformin after PSA progression on abiraterone did not affect further progression and had no meaningful clinical benefit [16].

A recent presentation of the MANSMED study (randomised single-blinded trial of metformin added to standard combined hormone treatment for men with either high-risk localised prostate cancer or metastatic castration-sensitive prostate cancer) demonstrated that patients receiving metformin had a longer time to castration-resistant disease (median 29 months, 95% CI 25 to 33) than those randomised to placebo (20 months, 95% CI 16 to 24, p = 0.01) [17]. This effect was most pronounced in men with high-risk localised disease and node-positive disease, marginal in those with low volume metastatic disease, and there was no benefit in those with high volume metastatic disease, and with the current follow-up interval, there was no difference in OS.

We found that metformin use showed associated OS and PSA response benefit only when co-administered with AAP, which is provocative. Whilst speculative, there are several potential mechanisms of action that may explain the improved OS and PSA-RR with metformin in this post-hoc analysis. AA resistance is in part driven by increased expression of the wild type androgen receptor (AR) and AR splice variants including AR variant 7 (AR-V7) [18]. *In vitro* studies have demonstrated that metformin may inhibit AR-V7 and that metformin in combination with AA may lead to increased cancer cell apoptosis [19]. Similar results were observed between metformin and the AR antagonist enzalutamide *in vitro* [20]. Preclinical evidence has also implicated the role of STAT3 signalling as a mechanism of resistance to abiraterone and enzalutamide, a pathway that may be inhibited by metformin via its effect on TGF-β [20]. These pre-clinical results may help to explain the more consistent benefits from metformin in those randomised to AAP than those randomised to placebo. However, our findings are limited by the small number of metformin users and the lack of control over which patients received metformin thus leading to potential confounding.

We found that statins were associated with a prolonged OS and improved PSA-RR in patients treated with AAP in COU-AA-301, who had previously received chemotherapy. This is in keeping with recent studies demonstrating improved OS among those treated with AAP and statin compared to statins alone [21-23]. However, we found no differences in OS in multivariate analysis or PSA response rate in patients who are chemotherapy-naïve and enrolled in the COU-AA-302 treated with AAP. Again, these findings are limited by the small number of statin users and the lack of control over which patients received statins thus leading to potential confounding.

There may be several reasons for the differences seen between COU-AA-301 and 302. The beneficial effects of statins on prostate cancer survival may be moderated by timing, dose and the duration of statin use. Some studies have shown improved OS among patients taking statins prior to diagnosis [24] while others found improved OS if statins were used after diagnosis [25]. Research has also demonstrated that not all statins are equal, with increased mortality benefit for hydrophilic statins compared to hydrophobic statins perhaps due to interference in lipid raft signalling or impacts on androgen availability [24]. Finally, higher doses appear to have greater effect on the prostatic epithelium [26]. Unfortunately, data regarding statin type, dose and the duration of therapy were unavailable but could possibly explain the differential findings between COU-AA-301 and 302.

There are important limitations to this study. This is exploratory hypothesis-generating post-hoc analysis of existing data and as such we chose not to perform statistical adjustments for false discovery rate. Several baseline imbalances existed between our medication groups that could affect our findings (Supplemental Tables 1 and 2). While adverse events by concurrent medication use are presented, we did not perform statistical comparisons due to the risk of false discovery. However, toxicities were similar between groups. We were not able to verify the causes of death which would inform whether metformin is improving prostate cancer-related death or simply decreasing other causes of death such as cardiovascular disease. Nonetheless, the association with OS in those treated with combination metformin and AAP remained significant in multivariate analysis after adjusting for these baseline imbalances, and metformin was associated with a prolonged OS when pooled across studies and treatment arms. While we demonstrate an improvement in OS and PSA response in some subgroups treated with metformin or statin, the results were inconsistent and require further study. In addition, radiographic progression-free survival data were unavailable for the cohorts involved, which may have provided correlative analyses. Finally, the subgroups of patients treated with metformin or statins are small, limiting our power to detect statistically significant differences and the takers of either drugs might simply be a surrogate for better health awareness, medical literacy and care, introducing a significant bias into the analyses.

Additional prospective studies with sufficient power to examine the effects of metformin and statins on outcomes in men with prostate cancer are needed, and several large studies are currently underway such as (i) metformin versus placebo for active surveillance (NCT01864096) (408 patients total), (ii) metformin in addition to standard of care in the mHSPC setting (STAMPEDE) (NCT00268476) (1800 patients), (iii) aspirin/atorvastatin in addition to standard of care in mCRPC (PEACE-4) (NCT03819101) (1210 patients), (iv) metformin in patients with mCRPC in combination with enzalutamide versus enzalutamide alone (SAKK0814) (NCT02640534) (169 patients).

## 5. Conclusion

Although methodologically limited, our results add to the growing body of evidence that metformin may prolong OS and increase PSA-RR among patients with mCRPC treated with AAP. While our findings regarding the effects of statins on OS differed between chemotherapy-treated and chemotherapy-naïve patients, they highlight the need for further prospective and controlled clinical trials regarding the adjunctive role that these medications may play for men with mCRPC.

## Supplementary Material

2

1

## Figures and Tables

**Table 1:** Median overall survival by study and by statin or metformin use.

	Metformin (median OS in months, 95% CI)	No metformin (median OS in months, 95% CI)	HR (95% CI)	p	Statin (median OS in months, 95% CI)	No statin (median OS in months, 95% CI)	HR (95% CI)	p
COU-AA-301								
AAP	19.4 (11.9-NR)	15.6 (14.7–16.9)	0.76 (0.55–1.05)	0.098	17.6 (15.1–19.6)	15.3 (14.3–16.7)	0.76 (0.63–0.93)	0.008
Placebo	14.0 (10.5–19.5)	11.1 (9.7–12.6)	0.85 (0.54–1.32)	0.47	13.2 (11.1–16.4)	10.7 (9.3–12.0)	0.81 (0.62–1.07)	0.13
COU-AA-302								
AAP	NR	NR	0.81 (0.48–1.36)	0.42	NR	NR	0.87 (0.63–1.21)	0.41
Placebo	NR	26.6 (25.8-NR)	0.68 (0.42–1.11	0.12	NR	27.2 (23.9-NR)	0.70 (0.52–0.96)	0.02

NR = not reached.

**Table 2:** Multivariate analyses comparing the use of statins or metformin in COU-AA-301 and COU-AA-302.

	Model including metformin			Model including statins		
COU-AA-301	Variable	HR (95% CI)	p	Variable	HR (95% CI)	p
**Abiraterone acetate plus Pred**						
	Metformin	0.71 (0.5–1.006)	0.054	Statin	0.76 (0.62–0.93)	0.0079
	LHRH2Trt	1.34 (1.1–1.6)	0.0026	LHRH2Trt	1.37 (1.13–1.65)	0.0012
	Liver mets	1.99 (1.54–2.58)	<0.0001	Liver mets	1.88 (1.45–2.43)	<0.0001
	ECOG >2	2.1 (1.6–2.7)	<0.0001	ECOG >2	2.09 (1.61–2.71)	<0.0001
	Albumin <4	1.50 (1.25–1.79)	<0.0001	Albumin <4	1.5 (1.26–1.81)	<0.0001
	LDH > ULN	2.24 (1.83–2.75)	<0.0001	LDH > ULN	2.27 (1.8–2.8)	<0.0001
	Alk Phos > ULN	1.35 (1.1–1.6)	0.003	Alk Phos > ULN	1.33 (1.09–1.62)	0.005
**Placebo**						
	Metformin	0.73 (0.46–1.15)	0.17	Statin	0.96 (0.72–1.27)	0.76
	LHRH2Trt	1.26 (0.98–1.63)	0.07	LHRH2Trt	1.25 (0.97–1.62)	0.08
	Liver mets	1.67 (1.06–2.63)	0.027	Liver mets	1.68 (1.07–2.65)	0.025
	ECOG >2	1.56 (1.06–2.30)	0.025	ECOG >2	1.59 (1.08–2.34)	0.019
	Albumin <4	2.24 (1.7–2.9)	<0.0001	Albumin <4	2.19 (1.68–2.86)	<0.0001
	LDH > ULN	1.99 (1.5–2.64)	<0.0001	LDH > ULN	2.01 (1.52–2.66)	<0.0001
	Alk Phos > ULN	1.71 (1.3–2.26)	0.0001	Alk Phos > ULN	1.68 (1.27–2.22)	0.0002
COU-AA-302	Variable	HR (95% CI)	p	Variable	HR (95% CI)	p
**Abiraterone acetate plus P**						
	Metformin	0.69 (0.48–0.98)	0.039	Statin	1.00 (0.8–1.2)	0.98
	LHRH2Trt	1.44 (1.16–1.79)	0.0011	LHRH2Trt	1.4 (1.13–1.75)	0.0022
	BPI 2-3	1.60 (1.25–2.04)	0.0001	BPI 2-3	1.63 (1.28–2.08)	<0.0001
	Age > 70	1.36 (1.1–1.7)	0.0054	Age > 70	1.35 (1.08–1.69)	0.0078
	Baseline PSA>39.5	1.57 (1.26–1.97)	<0.0001	Baseline PSA>39.5	1.57 (1.25–1.96)	<0.0001
	LDH > ULN	2.09 (1.58–2.76)	<0.0001	LDH > ULN	2.14 (1.62–2.83)	<0.0001
	>10 bone mets	2.01 (1.61–2.52)	<0.0001	>10 bone mets	1.98 (1.58–2.48)	<0.0001
**Placebo**						
	Metformin	0.66 (0.47–0.93)	0.018	Statin	0.88 (0.71–1.08)	0.22
	LHRH2Trt	1.69 (1.37–2.10)	<0.0001	LHRH2Trt	1.71 (1.38–2.12)	<0.0001
	BPI 2-3	1.23 (0.98–1.55)	0.067	BPI 2–3	1.24 (0.98–1.55)	0.066
	Age > 70	1.32 (1.07–1.63)	0.0085	Age > 70	1.37 (1.11–1.69)	0.0032
	Baseline PSA>39.5	1.43 (1.16–1.77)	0.0008	Baseline PSA>39.5	1.42 (1.14–1.75)	0.0014
	LDH > ULN	1.35 (1.02–1.79)	0.03	LDH > ULN	1.39 (1.05–1.84)	0.02
	>10 bone mets	1.45 (1.17–1.80)	0.0007	>10 bone mets	1.46 (1.18–1.81)	0.0006

LHRH2trt – Time from LHRH administration to baseline <36 months.

**Table 3:** PSA response stratified by study and by statin or metformin use.

	Metformin (% PSA response)	No metformin (% PSA response)	p	Statin (% PSA response)	No Statin (% PSA response)	p
COU-AA-301						
AAP	41.1%	28.6%	0.026	33.9%	28.0%	0.09
Placebo	3.2%	5.8%	0.55	4.8%	5.8%	0.71
COU-AA-302						
AAP	72.7%	60.0%	0.046	60.7%	62.1%	0.73
Placebo	27.9%	23.3%	0.41	23.3%	24.3%	0.78
