# Supplementary material for: Effects of metformin and statins on outcomes in men with castration-resistant metastatic prostate cancer: Secondary analysis of COU-AA-301 and COU-AA-302
Source: Eur J Cancer. Author manuscript; Available in PMC 2023 Feb 23. (PMC9949683; doi:10.1016/j.ejca.2022.03.042)
Supplement: 2 [file NIHMS1870978-supplement-2.docx]

**Supplemental Figure 1: Pooled effects of metformin on overall survival**


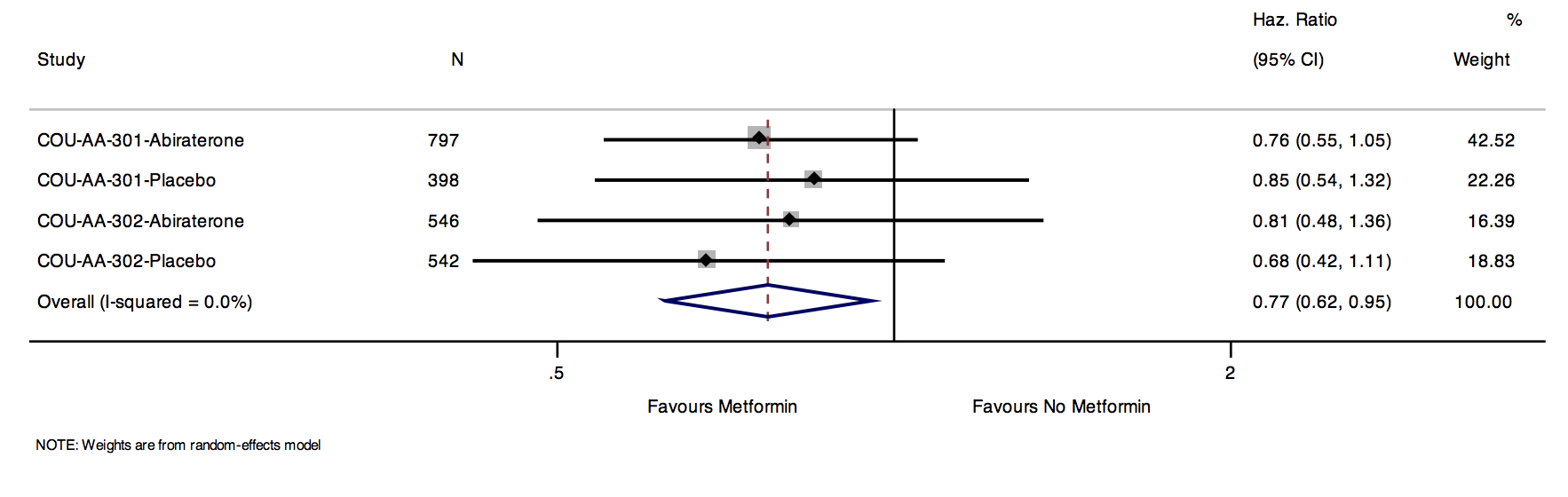


**Supplemental Figure 2: Pooled effects of Statins on overall survival**

**
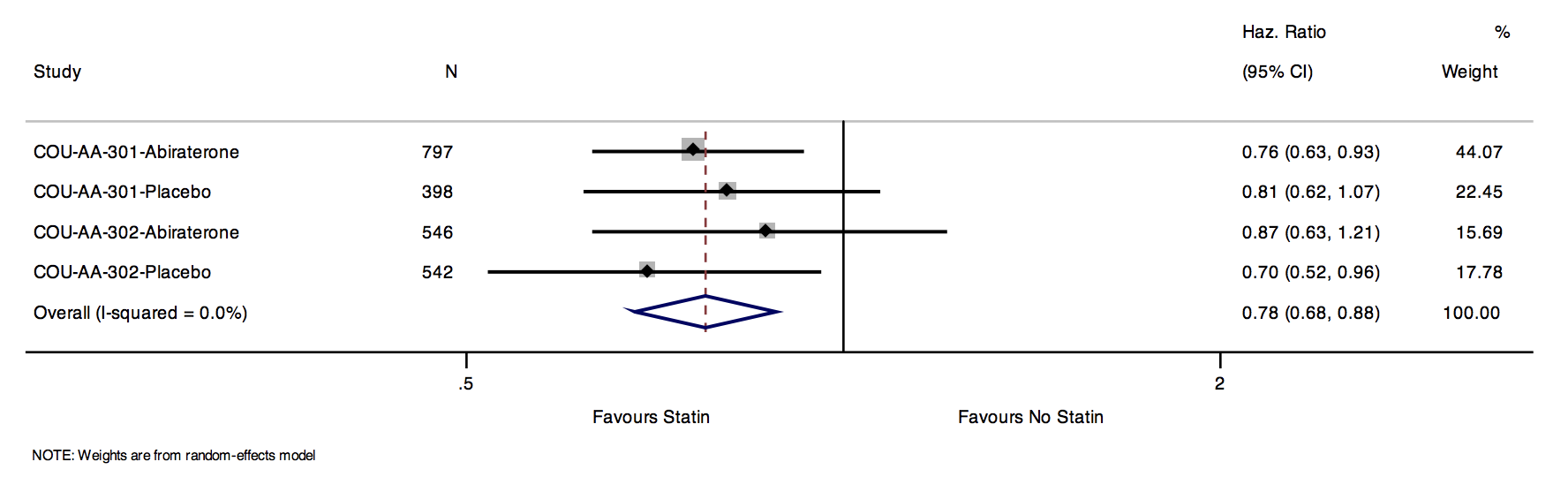
**
