# Supplementary material for: Effects of metformin and statins on outcomes in men with castration-resistant metastatic prostate cancer: Secondary analysis of COU-AA-301 and COU-AA-302
Source: Eur J Cancer. Author manuscript; Available in PMC 2023 Feb 23. (PMC9949683; doi:10.1016/j.ejca.2022.03.042)
Supplement: 1 [file NIHMS1870978-supplement-1.docx]

**Supplemental Table 1: Baseline Characteristics Stratified by metformin and Statin Use in the COU-AA-301 Study**

|  | Metformin  N (%) or  Mean (SD) | No metformin  N (%) or  Mean (SD) | P value | Statin  N (%) or  Mean (SD) | No Statin  N (%) or  Mean(SD) | P value |
| --- | --- | --- | --- | --- | --- | --- |
| AAP Group | N=73 | N=724 |  | N=236 | N=561 |  |
| Age Group  <65  65-74  75-84  >85 | 17 (23.3%)  32 (43.8%)  22 (30.2%)  2 (2.7%) | 215 (29.7%)  313 (42.2%)  179 (24.7%)  17 (2.4%) | 0.62 | 47 (19.9%)  110 (46.6%)  67 (28.4%)  12 (5.1%) | 185 (33.0%)  235 (41.9%)  134 (23.9%)  7 (1.2%) | 0.0001 |
| ECOG  0  1  2 | 19 (26.0%)  40 (54.8%)  14 (19.2%) | 255 (35.2%)  401 (55.4%)  68 (9.4%) | 0.02 | 83 (35.2%)  121 (51.3%)  32 (13.6%) | 191 (34.1%)  320 (57.0%)  50 (8.9%) | 0.10 |
| Pain at Baseline  0-1  2-3  >3 | 25 (34.2%)  23 (31.5%)  25 (34.3%) | 251 (35.4%)  226 (31.9%)  232 (32.7%) | 0.96 | 82 (34.9%)  79 (33.6%)  74 (31.5%) | 194 (35.5%)  170 (31.1%)  183 (33.4%) | 0.76 |
| Cardiovascular Disease  YES  NO | 63 (86.3%)  10 (13.7%) | 475 (65.6%)  249 (34.4%) | <0.001 | 202 (85.6%)  34 (14.4%) | 336 (59.9%)  225 (40.1%) | <0.001 |
| Gleason Score  2-4  5-7  8-10 | 1 (1.5%)  33 (50.0%)  32 (48.5%) | 8 (1.3%)  299 (47.4%)  324(51.3%) | 0.9 | 3 (1.4%)  109 (51.9%)  98 (46.7%) | 6 (1.2%)  223 (45.8%)  258 (53.0%) | 0.3 |
| Tumour Stage  T0  T1  T2  T3  T4  UNK  NA | 1 (1.6%)  11 (15.7%)  23 (32.9%)  21 (30.0%)  3 (4.3%)  8 (11.4%)  3 (4.3%) | 0  79 (11,4%)  178 (25.6%)  261 (37.6%)  53 (7.6%)  94 (13.5%)  29 (4.2%) | 0.026 | 1 (0.4%)  30 (13.3%)  69 (30.7%)  78 (34.7%)  11 (4.9%)  29 (12.9%)  7 (3.1%) | 0  60 (11.1%)  132 (24.5%)  204 (37.8%)  45 (8.3%)  73 (13.5%)  25 (4.6%) | 0.16 |
| Nodal Stage  N0  N1  N2  N3  UNK  NA | 33 (49.3%)  5 (7.5%)  3 (4.5%)  0  22 (32.8%)  4 (6.0%) | 309 (44.8%)  81 (11.8%)  23 (3.3%)  5 (0.7%)  220 (31.9%)  51 (7.4%) | 0.83 | 112 (50.7%)  21 (9.5%)  8 (3.6%)  1 (0.5%)  58 (26.2%)  21 (9.5%) | 230 (43.0%)  65 (12.1%)  18 (3.4%)  4 (0.7%)  184 (34.4%)  34 (6.4%) | 0.12 |
| Metastases Stage  M0  M1  UNK  NA | 25 (37.3%)  21 (41.2%)  16 (23.9%)  5 (7.5%) | 322 (57.7%)  207 (37.1%)  139 (19.9%)  29 (4.2%) | 0.38 | 111 (50.2%)  45 (20.4%)  54 (24.4%)  11 (5.0%) | 236 (43.5%)  183 (33.7%)  101 (18.6%)  23 (4.2%) | 0.003 |
| Progressive Disease  PSA only  Radiographic | 20 (27.4%)  53 (72.6%) | 218 (30.1%)  506 (69.9%) | 0.6 | 77 (32.6%)  159 (67.4%) | 161 (28.7%)  400 (71.3%) | 0.27 |
| Disease sites  Bone  Soft Tissue  Bone & Soft Tissue | 31 (43.1%)  5 (6.9%)  36 (50.0%) | 268 (37.4%)  74 (10.7%)  375 (52.3%) | 0.50 | 99 (41.9%)  19 (8.1%)  118 (50.0%) | 200 (36.2%)  60 (10.8%)  293 (53.0%) | 0.22 |
| Liver metastases  Yes  No | 11 (15.1%)  62 (84.9%) | 78 (10.8%)  646 (89.2%) | 0.27 | 21 (8.9%)  215 (91.1%) | 68 (12.1%)  493 (87.9%) | 0.19 |
| LDH  >ULN  ≤ ULN | 21 (28.8%)  52 (71.2%) | 281 (39.6%)  429 (60.4%) | 0.07 | 76 (32.6%)  157 (67.4%) | 226 (41.1%)  324 (58.9%) | 0.03 |
| Albumin  ≤4  >4 | 33 (45.2%)  40 (54.8%) | 360 (50.2%)  357 (49.8%) | 0.41 | 121 (51.5%)  114 (48.5%) | 272 (49.0%)  283 (51.0%) | 0.52 |
| Alkaline Phosphatase  >ULN  ≤ULN | 26 (35.6%)  47 (64.4%) | 307 (42.8%)  410 (57.2%) | 0.23 | 85 (36.2%)  150 (63.8%) | 248 (44.7%)  307 (55.3%) | 0.03 |
| Short time on LHRH  ≤36 months  Other | 21 (29.2%)  51 (70.8%) | 217 (30.6%)  493 (69.4%) | 0.81 | 69 (29.6%)  164 (70.4%) | 169 (30.8%)  380 (69.2%) | 0.74 |
| Body Mass Index | (n=68)  29.0 +/- 5.2 | (n=682)  27.3 +/- 4.6 | 0.004 | (n=222)  28.5 +/- 5.5 | (n=529)  27.0 +/- 4.2 | <0.001 |
|  |  |  |  |  |  |  |
| Placebo Group | N=31 | N=367 |  | N=103 | N=295 |  |
| Age Group  <65  65-74  75-84  >85 | 10 (38.7%)  12 (29.0%)  9 (32.3%)  0 | 109 (29.8%)  155 (42.2%)  95 (26.0%)  7 (1.9%) | 0.84 | 23 (22.3%)  47 (45.6%)  31 (30.1%)  2 (1.9%) | 96 (32.7%)  120 (40.8%)  73 (24.8%)  5 (1.7%) | 0.27 |
| ECOG  0  1  2 | 9 (29.0%)  19 (61.3%)  3 (9.7%) | 126 (34.3%)  199 (54.2%)  42 (11.4%) | 0.75 | 37 (35.9%)  55 (53.4%)  11 (10.7%) | 98 (33.2%)  163 (55.3%)  34 (11.5%) | 0.88 |
| Pain at Baseline  0-1  2-3  >3 | 13 (43.3%)  8 (26.7%)  9 (30.0%) | 137 (38.4%)  102 (28.6%)  118 (33.0%) | 0.87 | 38 (37.6%)  32 (31.7%)  31 (30.7%) | 112 (39.2%)  78 (27.3%)  96 (33.6%) | 0.69 |
| Cardiovascular Disease  Yes  No | 26 (83.9%)  5 (16.1%) | 249 (67.9%)  118 (32.2%) | 0.06 | 88 (85.4%)  15 (14.6%) | 187 (63.4%)  108 (36.6%) | <0.001 |
| Gleason Score  2-4  5-7  8-10 | 0  13 (50.0%)  13 (50.0%) | 5 (1.5%)  143 (44.1%)  176 (54.3%) | 0.71 | 1 (1.1%)  49 (53.8%)  41 (45.1%) | 4 (1.5%)  107 (41.3%)  148 (57.1%) | 0.12 |
| Tumour Stage  T0  T1  T2  T3  T4  UNK  NA | 0  3 (12.0%)  11 (44.0%)  7 (28.0%)  1 (4.0%)  5 (16.7%)  3 (10.0%) | 0  37 (12.0%)  88 (28.5%)  129 (41.7%)  30 (9.7%)  50 (13.9%)  25 (7.0%) | 0.52 | 0  7 (6.8%)  29 (28.2%)  38 (36.9%)  8 (7.8%)  13 (12.6%)  8 (7.8%) | 0  33 (11.5%)  70 (24.5%)  98 (34.3%)  23 (8.0%)  42 (14.7%)  20 (7.0%) | 0.77 |
| Nodal Stage  N0  N1  N2  N3  UNK  NA | 12 (57.1%)  4 (19.0%)  0  1 (4.8%)  9 (30.0%)  4 (13.3%) | 141 (60.5%)  42 (18.0%)  12 (5.2%)  6 (2.6%)  123 (34.6%)  32 (9.0%) | 0.82 | 49 (47.6%)  13 (12.6%)  3 (2.9%)  2 (1.9%)  27 (26.2%)  9 (8.7%) | 104 (36.7%)  33 (11.7%)  9 (3.2%)  5 (1.8%)  105 (37.1%)  27 (9.5%) | 0.42 |
| Metastases Stage  M0  M1  UNK  NA | 13 (56.5%)  6 (26.1%)  7 (23.3%)  4 (13.3%) | 158 (54.5%)  115 (39.7%)  68 (19.0%)  17 (4.7%) | 0.15 | 47 (45.6%)  29 (28.2%)  20 (19.4%)  7 (6.8%) | 124 (43.5%)  92 (32.3%)  55 (19.3%)  14 (4.9%) | 0.8 |
| Progressive Disease  PSA only  Radiographic | 8 (25.8%)  23 (74.2%) | 117 (31.9%)  250 (68.1%) | 0.48 | 36 (35.0%)  67 (65.0%) | 89 (30.2%)  206 (69.8%) | 0.37 |
| Disease sites  Bone  Soft Tissue  Bone & Soft Tissue | 13 (41.9%)  4 (12.9%)  14 (45.2%) | 162 (44.6%)  32 (8.8%)  169 (46.6%) | 0.75 | 56 (54.4%)  14 (13.6%)  33 (32.0%) | 119 (40.9%)  22 (7.6%)  150 (51.5%) | 0.002 |
| Liver metastases  Yes  No | 4 (12.9%)  27 (87.1%) | 25 (6.8%)  342 (93.2%) | 0.21 | 6 (5.8%)  97 (94.2%) | 23 (7.8%)  272 (92.2%) | 0.51 |
| LDH  >ULN  ≤ ULN | 11 (36.7%)  19 (63.3%) | 157 (44.1%)  199 (55.9%) | 0.43 | 39 (39.0%)  61 (61.0%) | 129 (45.1%)  157 (54.9%) | 0.29 |
| Albumin  ≤4  >4 | 15 (48.4%)  16 (51.6%) | 168 (46.5%)  193 (53.5%) | 0.84 | 41 (40.6%)  60 (59.4%) | 142 (48.8%)  149 (51.2%) | 0.16 |
| Alkaline Phosphatase  >ULN  ≤ULN | 13 (41.9%)  18 (58.1%) | 158 (43.8%)  203 (56.2%) | 0.84 | 33 (32.7%)  68 (67.3%) | 138 (47.4%)  153 (52.6%) | 0.01 |
| Short time on LHRH  ≤36 months  Other | 10 (33.3%)  20 (66.7%) | 122 (33.8%)  239 (66.2%) | 0.96 | 31 (30.4%)  71 (69.6%) | 101 (34.9%)  188 (65.1%) | 0.40 |
| Body Mass Index (mean +/- SD) | (n=31)  29.0 +/- 5.4 | (n=350)  27.4 +/- 4.6 | 0.07 | (n=102)  28.0 +/- 4.4 | (n=279)  27.4 +/- 4.7 | 0.23 |

Note: where the total number of patients does not add up to the total in the group, the remaining data was missing

**Supplemental Table 2: Baseline Characteristics Stratified by metformin and Statin Use in the COU-AA-302 Study**

|  | Metformin  N (%) | No Metformin  N (%) | P value | Statin  N (%) | No Statin  N (%) | P value |
| --- | --- | --- | --- | --- | --- | --- |
| AAP Group | N=66 | N=480 |  | N=229 | N=317 |  |
| Age Group  <65  65-74  75-84  >85 | 14 (21.3%)  34 (51.5%)  16 (24.2%)  2 (3.0%) | 121 (25.2%)  192 (40.0%)  141 (29.4%)  26 (5.4%) | 0.33 | 42 (18.3%)  103 (45.0%)  73 (31.9%)  11 (4.8%) | 93 (29.3%)  123 (38.8%)  84 (26.5%)  17 (5.4%) | **0.027** |
| ECOG  0  1 | 46 (69.7%)  20 (30.3%) | 370 (77.1%)  110 (22.9%) | 0.19 | 177 (77.3%)  52 (22.7%) | 239 (75.4%)  78 (24.6%) | 0.61 |
| Pain at Baseline  0-1  2-3  >3 | 47 (72.3%)  12 (21.5%)  4 (6.2%) | 357 (75.6%)  93 (19.7%)  22 (4.7%) | 0.8 | 167 (73.2%)  52 (22.8%)  9 (3.9%) | 237 (76.7%)  55 (17.8%)  17 (5.5%) | 0.28 |
| Cardiovascular Disease  Yes  No | 61 (92.4%)  5 (7.6%) | 334 (69.6%)  146 (30.4%) | <0.0001 | 199 (86.9%)  30 (13.1%) | 196 (61.8%)  121 (38.2%) | <0.001 |
| Gleason Score  2-4  5-7  8-10 | 0  23 (39.0%)  36 (61.0%) | 6 (1.4%)  196 (45.7%)  227 (52.9%) | 0.37 | 4 (1.9%)  105 (50.5%)  99 (47.6%) | 2 (0.7%)  114 (40.7%)  164 (58.6%) | 0.036 |
| Tumour Stage  T1  T2  T3  T4  TX  UNK  NA | 9 (13.6%)  20 (30.3%)  22 (33.3%)  4 (6.1%)  5 (7.6%)  6 (9.1%)  0 | 56 (11.8%)  131 (27.6%)  151 (31.8%)  27 (5.7%)  37 (7.8%)  71 (14.9%)  2 (0.4%) | 0.91 | 32 (14.0%)  66 (28.8%)  76 (33.2%)  13 (5.7%)  11 (4.8%)  31 (13.5%)  0 | 33 (10.6%)  85 (27.2%)  97 (31.1%)  18 (5.8%)  31 (9.9%)  46 (14.7%)  2 (0.6%) | 0.26 |
| Nodal Stage  N0  N1  N2  N3  NX  UNK  NA | 34 (51.5%)  7 (10.6%)  1 (1.5%)  1 (1.5%)  11 (16.7%)  12 (18.2%)  0 | 184 (38.7%)  54 (11.3%)  15 (3.2%)  7 (1.5%)  107 (22.5%)  105 (22.1%)  4 (0.8%) | 0.57 | 102 (44.5%)  28 (12.2%)  6 (2.6%)  2 (0.9%) | 116 (37.1%)  33 (10.5%)  10 (3.2%)  6 (1.9%) | 0.51 |
| Metastases Stage  M0  M1  MX  UNK  NA | 36 (54.5%)  14 (21.2%)  6 (9.1%)10 (15.2%)  0 | 203 (42.6%)  121 (25.4%)  69 (14.5%)  81 (17.0%)  2 (0.4%) | 0.42 | 111 (48.5%)  42 (18.3%)  36 (15.7%)  39 (17.0%)  1 (0.4%) | 128 (40.9%)  93 (29.7%)  39 (12.5%)  52 (16.6%)  1 (0.3%) | 0.046 |
| Disease sites  Bone  Soft Tissue  Bone & Soft Tissue | 33 (50%)  9 (13.6%)  24 (36.4%) | 244 (51%)  83 (17.4%)  151 (31.6%) | 0.64 | 111 (48.5%)  42 (18.3%)  76 (33.2%) | 166 (52.7%)  50 (15.9%)  99 (31.4%) | 0.586 |
| Bone metastases  ≥10  <10 | 25 (37.9%)  41 (62.1%) | 168 (35.1%)  310 (64.9%) | 0.66 | 77 (33.6%)  152 (66.4%) | 116 (36.8%)  199 (63.2%) | 0.44 |
| LDH  >ULN  ≤ ULN | 4 (6.1%)  62 (93.9%) | 75 (15.7%)  402 (84.3%) | 0.04 | 36 (15.7%)  193 (84.3%) | 43 (13.7%)  271 (86.3%) | 0.51 |
| Short time on LHRH  ≤ 36 months  Other | 32 (48.5%)  34 (51.5%) | 197 (42.5%)  278 (58.5%) | 0.28 | 93 (40.6%)  136 (59.4%) | 136 (43.6%)  176 (56.4%) | 0.49 |
| High PSA  PSA > median (39.5)  Other | 30 (45.5%)  36 (54.5%) | 252 (52.5%)  228 (47.5%) | 0.28 | 106 (46.3%)  123 (53.7%) | 176 (55.5%)  141 (44.5%) | 0.03 |
| BPI category  BPI 0-1  BPI 2-3 | 47 (77.0%)  14 (23.0%) | 323 (73.7%)  115 (26.3%) | 0.58 | 158 (75.2%)  52 (24.8%) | 212 (73.4%)  77 (26.6%) | 0.64 |
| Body Mass Index (mean +/- SD) | (n=61)  30.4 +/- 3.8 | (n=460)  28.7 +/- 4.3 | 0.005 | (n=220)  29.8 +/- 4.1 | (n=301)  28.3 +/- 4.3 | <0.001 |
|  |  |  |  |  |  |  |
| Placebo Group | N=68 | N=474 |  | N=207 | N=335 |  |
| Age Group  <65  65-74  75-84  >85 | 18 (26.5%)  34 (50.0%)  12 (17.6%)  4 (5.9%) | 137 (28.9%)  188 (39.7%)  124 (26.2%)  25 (5.3%) | 0.33 | 45 (21.7%)  85 (41.1%)  65 (31.4%)  12 (5.8%) | 110 (32.8%)  137 (40.9%)  71 (21.2%)  17 (5.1%) | 0.012 |
| ECOG  0  1 | 54 (79.4%)  12 (20.6%) | 360 (75.9%)  114 (24.1%) | 0.53 | 148 (71.5%)  59 (28.5%) | 266 (79.4%)  69 (20.6%) | 0.035 |
| Pain at Baseline  0-1  2-3  >3 | 48 (73.8%)  10 (15.4%)  7 (10.8%) | 334 (72%)  111 (23.9%)  10 (4.1%) | 0.03 | 140 (69.7%)  45 (22.4%)  16 (8.0%) | 242 (73.8%)  76 (23.2%)  10 (3.0%) | 0.04 |
| Cardiovascular Disease  Yes  No | 58 (85.3%)  10 (14.7%) | 333 (70.3%)  141(29.7%) | 0.01 | 181 (87.4%)  26 (12.6%) | 210 (62.7%)  125 (37.3%) | <0.0001 |
| Gleason Score  2-4  5-7  8-10 | 0  36 (57.1%)  27 (42.9%) | 6 (1.3%)  212 (47.6%)  227 (51.0%) | 0.27 | 3 (1.5%)  92 (47.2%)  100 (51.3%) | 3 (1.0%)  156 (49.8%)  154 (49.2%) | 0.73 |
| Tumour Stage  T0  T1  T2  T3  T4  TX  UNK  NA | 1 (1.5%)  6 (8.8%)  21 (30.9%)  23 (33.8%)  4 (5.9%)  5 (7.4%)  8 (11.8%)  0 | 1 (0.2%)  65 (13.7%)  128 (27.1%)  139 (29.4%)  35 (7.4%)  30 (6.3%)  71 (15.0%)  4 (0.8%) | 0.58 | 1 (0.5%)  29 (14.0%)  59 (28.5%)  60 (29.0%)  13 (6.3%)  11 (5.3%)  32 (15.5%)  2 (1.0%) | 1 (0.3%)  42 (12.6%)  90 (26.9%)  102 (30.5%)  26 (7.8%)  24 (7.2%)  47 (14.2%)  2 (0.6%) | 0.96 |
| Nodal Stage  N0  N1  N2  N3  NX  UNK  NA | 28 (41.8%)  7 (10.4%)  1 (1.5%)  1 (1.5%)  15 (22.4%)  13 (19.4%)  2 (3.0%) | 192 (40.6%)  51 (10.8%)  9 (1.9%)  7 (1.5%)  99 (20.9%)  108 (22.8%)  7 (1.5%) | 0.97 | 92 (44.7%)  21 (10.2%)  3 (1.5%)  3 (1.5%)  39 (18.9%)  42 (20.4%)  6 (2.9%) | 128 (38.3%)  37 (11.1%)  7 (2.1%)  5 (1.5%)  75 (22.5%)  79 (23.7%)  3 (0.9%) | 0.41 |
| Metastases Stage  M0  M1  MX  UNK  NA | 30 (44.1%)  15 (22.1%)  14 (20.6%)  8 (11.8%)  1 (1.5%) | 200 (42.3%)  127 (26.8%)  74 (15.6%)  67 (14.2%)  5 (1.1%) | 0.77 | 102 (49.3%)  42 (20.3%)  30 (14.5%)  28 (13.5%)  5 (2.4%) | 128 (38.3%)  100 (29.9%)  58 (17.4%)  47 (14.1%)  1 (0.3%) | 0.007 |
| Disease sites  Bone  Soft Tissue  Bone & Soft Tissue | 34 (50%)  18 (26.5%)  16 (23.5%) | 237 (50%)  92 (19.4%)  145 (30.6%) | 0.29 | 102 (49.3%)  46 (22.2%)  59 (28.5%) | 169 (50.4%)  64 (19.1%)  102 (30.4%) | 0.67 |
| Bone metastases  ≥10  <10 | 25 (36.8%)  43 (63.2%) | 163 (34.4%)  311 (65.6%) | 0.70 | 68 (32.9%)  139 (67.1%) | 120 (35.8%)  215 (64.2%) | 0.48 |
| LDH  >ULN  ≤ ULN | 4 (5.9%)  64 (94.1%) | 76 (16.2%)  392 (83.8%) | 0.025 | 27 (13.2%)  177 (86.8%) | 53 (16.0%)  279 (84.0%) | 0.39 |
| Short time on LHRH  ≤ 36 months  Other | 22 (32.4%)  46 (67.6%) | 210 (44.5%)  262 (55.5%) | 0.06 | 86 (41.5%)  121 (58.5%) | 146 (43.8%)  187 (56.2%) | 0.60 |
| High PSA  PSA > median (39.5)  Other | 33 (48.5%)  35 (51.5%) | 227 (48.2%)  244 (51.8%) | 0.96 | 86 (41.7%)  120 (58.3%) | 174 (52.3%)  159 (47.7%) | 0.018 |
| BPI category  BPI 0-1  BPI 2-3 | 44 (77.2%)  13 (22.8%) | 302 (69.3%)  134 (30.7%) | 0.22 | 135 (73.0%)  50 (27.0%) | 211 (68.5%)  97 (31.5%) | 0.29 |
| Body Mass Index (mean +/- SD) | (n=62)  30.9 +/- 4.9 | (n=452)  28.8 +/- 4.6 | 0.001 | (n=198)  30.1 +/- 4.8 | (n=316)  28.4 +/- 4.6 | <0.001 |

Note: where the total number of patients does not add up to the total in the group, the remaining data was missing

**Supplemental Table 3: Toxicity rates in COU-AA-301 among Statin users and non-users in the placebo and Abiraterone arms**

|  | Statin (n= 339) | | | | No Statin (n=846) | | | |
| --- | --- | --- | --- | --- | --- | --- | --- | --- |
|  | **AAP (n=236)** | | **Placebo (n=103)** | | **AAP (n=555)** | | **Placebo (n=291)** | |
| Toxicity | **Total (%)** | **Grade 3/4** | **Total** | **Grade 3/4** | **Total** | **Grade 3/4** | **Total** | **Grade 3/4** |
| Anemia | 73 (30.9%) | 22 (9.3%) | 25 (24.3%) | 7 (6.8%) | 138 (24.9%) | 45 (8.1%) | 87 (29.9%) | 25 (8.6%) |
| Thrombocytopenia | 10 (4.2%) | 4 (1.7%) | 3 (2.9%) | 1 (1%) | 21 (3.8%) | 7 (1.3%) | 12 (4.1%) | 1 (0.3%) |
| Neutropenia | 4 (1.7%) | 1 (0.4%) | 1 (1.0%) | 0 (0%) | 6 (1.1%) | 1 (0.2%) | 1 (0.3%) | 1 (0.3%) |
| Febrile neutropenia | 1 (0.4%) | 1 (0.4%) | 0 | 0 (0%) | 2 (0.4%) | 2 (0.4%) | 0 | 0 (0%) |
| Diarrhea | 57 (24.2%) | 5 (2.1%) | 18 (17.5%) | 2 (1.9%) | 112 (20.2%) | 5 (0.9%) | 41 (14.1%) | 3 (1%) |
| Fatigue | 126 (53.4%) | 23 (9.7%) | 48 (46.6%) | 12 (11.7%) | 260 (46.8%) | 56 (10.1%) | 127 (43.6%) | 30 (10.3%) |
| Asthenia | 41 (17.4%) | 9 (3.8%) | 14 (13.6%) | 3 (2.9%) | 93 (16.8%) | 18 (3.2%) | 41 (14.1%) | 5 (1.7%) |
| Back pain | 92 (39.0%) | 24 (10.2%) | 39 (37.9%) | 11 (10.7%) | 191 (34.4%) | 36 (6.5%) | 103 (35.4%) | 30 (10.3%) |
| Nausea | 81 (34.3%) | 11 (4.7%) | 34 (33.0%) | 1 (1%) | 192 (34.6%) | 9 (1.6%) | 99 (34.0%) | 10 (3.4%) |
| Vomiting | 68 (28.8%) | 8 (3.4%) | 27 (26.2%) | 2 (1.9%) | 141 (25.4%) | 14 (2.5%) | 75 (25.8%) | 11 (3.8%) |
| Hematuria | 25 (10.6%) | 3 (1.3%) | 11 (10.7%) | 3 (2.9%) | 55 (9.9%) | 10 (1.8%) | 23 (7.9%) | 6 (2.1%) |
| Abdominal pain | 42 (17.8%) | 6 (2.5%) | 15 (14.6%) | 2 (1.9%) | 72 (13.0%) | 14 (2.5%) | 32 (11.0%) | 6 (2.1%) |
| Pain in extremity | 50 (21.2%) | 11 (4.7%) | 14 (13.6%) | 4 (3.9%) | 122 (22.0%) | 17 (3.1%) | 71 (24.4%) | 18 (6.2%) |
| Dyspnea | 40 (16.9%) | 7 (3%) | 20 (19.4%) | 3 (2.9%) | 83 (15.0%) | 12 (2.2%) | 31 (10.7%) | 6 (2.1%) |
| Constipation | 71 (30.1%) | 5 (2.1%) | 37 (35.9%) | 1 (1%) | 166 (29.9%) | 6 (1.1%) | 89 (30.6%) | 4 (1.4%) |
| Pyrexia | 26 (11.0%) | 2 (0.8%) | 11 (10.7%) | 1 (1%) | 65 (11.7%) | 2 (0.4%) | 25 (8.6%) | 4 (1.4%) |
| Arthralgia | 86 (36.4%) | 21 (8.9%) | 31 (30.1%) | 5 (4.9%) | 162 (29.2%) | 25 (4.5%) | 64 (22.0%) | 12 (4.1%) |
| Urinary tract infection | 38 (16.1%) | 10 (4.2%) | 6 (5.8%) | 1 (1%) | 73 (13.2%) | 16 (2.9%) | 24 (8.2%) | 2 (0.7%) |
| Pain | 13 (5.5%) | 1 (0.4%) | 3 (2.9%) | 0 (0%) | 27 (4.9%) | 7 (1.3%) | 18 (6.2%) | 8 (2.7%) |
| Bone pain | 61 (25.8%) | 11 (4.7%) | 34 (33.0%) | 7 (6.8%) | 165 (29.7%) | 41 (7.4%) | 83 (28.5%) | 23 (7.9%) |
| Peripheral oedema | 1 (0.4%) | 0 (0%) | 1 (1.0%) | 0 (0%) | 156 (28.1%) | 13 (2.3%) | 56 (19.2%) | 3 (1%) |
| Hypokalemia | 54 (22.9%) | 16 (6.8%) | 10 (9.7%) | 1 (1%) | 99 (17.8%) | 24 (4.3%) | 26 (8.9%) | 2 (0.7%) |
| Cardiac disorder | 55 (23.3%) | 20 (8.5%) | 13 (12.6%) | 2 (1.9%) | 67 (12.1%) | 21 (3.8%) | 21 (7.2%) | 5 (1.7%) |
| Liver function test abnormalities | 0 (0%) | 0 (0%) |  | 0 (0%) | 0 | 0 (0%) | 1 (0.3%) | 1 (0.3%) |
| Hypertension | 29 (12.3%) | 4 (1.7%) | 5 (4.9%) | 1 (1%) | 53 (9.5%) | 9 (1.6%) | 22 (7.6%) | 0 (0%) |

**Supplemental Table 4: Toxicity rates in COU-AA-302 among Statin users and non-users in the placebo and Abiraterone arms**

|  | Statin (n= 436) | | | | No Statin (n=646) | | | |
| --- | --- | --- | --- | --- | --- | --- | --- | --- |
|  | **AAP (n=229)** | | **Placebo (n=227)** | | **AAP (n=313)** | | **Placebo (n=333)** | |
| Toxicity | **Total (%)** | **Grade 3/4** | **Total** | **Grade 3/4** | **Total** | **Grade 3/4** | **Total** | **Grade 3/4** |
| Anemia | 29(12.7%) | 8(3.5%) | 27(13%) | 6(2.9%) | 34 (10.9%) | 7(2.2%) | 26 (7.8%) | 4(1.2%) |
| Thrombocytopenia | 5(2.2%) | 1(0.4%) | 3(1.4%) | 1(0.5%) | 6 (1.9%) | 1(0.3%) | 5 (1.5%) | 3(0.9%) |
| Neutropenia | 2(0.9%) | 1(0.4%) | 2(1%) | 1(0.5%) | 1 (0.3%) | 1(0.3%) | 2 (0.6%) | 1(0.3%) |
| Febrile neutropenia | 0(0%) | 0(0%) | 1(0.5%) | 1(0.5%) | 0 | 0(0%) | 0 | 0(0%) |
| Diarrhea | 58(25.3%) | 3(1.3%) | 48(23.2%) | 0(0%) | 79 (25.2%) | 5(1.6%) | 50 (15.0%) | 5(1.5%) |
| Fatigue | 111(48.5%) | 9(3.9%) | 88(42.5%) | 5(2.4%) | 133 (42.5%) | 7(2.2%) | 112 (33.6%) | 7(2.1%) |
| Asthenia | 22(9.6%) | 1(0.4%) | 23(11.1%) | 3(1.4%) | 24 (7.7%) | 0(0%) | 24 (7.2%) | 3(0.9%) |
| Back pain | 83(36.2%) | 5(2.2%) | 78(37.7%) | 9(4.3%) | 117 (37.4%) | 13(4.2%) | 103 (30.9%) | 12(3.6%) |
| Nausea | 60(26.2%) | 4(1.7%) | 55(26.6%) | 0(0%) | 84 (26.8%) | 2(0.6%) | 72 (21.6%) | 1(0.3%) |
| Vomiting | 43(18.8%) | 1(0.4%) | 27(13%) | 0(0%) | 44 (14.1%) | 3(1%) | 34 (10.2%) | 0(0%) |
| Hematuria | 28(12.2%) | 3(1.3%) | 18(8.7%) | 2(1%) | 34 (10.9%) | 4(1.3%) | 15 (4.5%) | 1(0.3%) |
| Abdominal pain | 23(10%) | 2(0.9%) | 21(10.1%) | 4(1.9%) | 29 (9.3%) | 3(1%) | 28 (8.4%) | 5(1.5%) |
| Pain in extremity | 44(19.2%) | 4(1.7%) | 45(21.7%) | 4(1.9%) | 63 (20.1%) | 0(0%) | 44 (13.2%) | 1(0.3%) |
| Dyspnea | 37(16.2%) | 10(4.4%) | 24(11.6%) | 2(1%) | 35 (11.2%) | 5(1.6%) | 31 (9.3%) | 3(0.9%) |
| Constipation | 69(30.1%) | 0(0%) | 51(24.6%) | 0(0%) | 75 (24.0%) | 2(0.6%) | 61 (18.3%) | 3(0.9%) |
| Pyrexia | 21(9.2%) | 2(0.9%) | 11(5.3%) | 0(0%) | 36 (11.5%) | 1(0.3%) | 23 (6.9%) | 1(0.3%) |
| Arthralgia | 77(33.6%) | 7(3.1%) | 58(28%) | 4(1.9%) | 96 (30.7%) | 4(1.3%) | 74 (22.2%) | 6(1.8%) |
| Urinary tract infection | 20(8.7%) | 7(3.1%) | 21(10.1%) | 3(1.4%) | 33 (10.5%) | 5(1.6%) | 21 (6.3%) | 0(0%) |
| Pain | 10(4.4%) | 0(0%) | 6(2.9%) | 0(0%) | 17 (5.4%) | 0(0%) | 11 (3.3%) | 3(0.9%) |
| Bone pain | 60(26.2%) | 4(1.7%) | 43(20.8%) | 6(2.9%) | 87 (27.8%) | 7(2.2%) | 73 (21.9%) | 7(2.1%) |
| Peripheral oedema | 69(30.1%) | 3(1.3%) | 53(25.6%) | 1(0.5%) | 80 (25.6%) | 0(0%) | 66 (19.8%) | 4(1.2%) |
| Hypokalemia | 57(24.9%) | 9(3.9%) | 28(13.5%) | 6(2.9%) | 44 (14.1%) | 5(1.6%) | 41 (12.3%) | 4(1.2%) |
| Cardiac disorder | 47(20.5%) | 20(8.7%) | 39(18.8%) | 6(2.9%) | 55 (17.6%) | 10(3.2%) | 42 (12.6%) | 7(2.1%) |
| Liver function test abnormalities | 2(0.9%) | 0(0%) | 0(0%) | 0(0%) | 2 (0.6%) | 0(0%) | 0 | 0(0%) |
| Hypertension | 58(25.3%) | 14(6.1%) | 29(14%) | 9(4.3%) | 71 (22.7%) | 11(3.5%) | 45 (13.5%) | 8(2.4%) |

**Supplemental Table 5: Toxicity rates in COU-AA-301 among metformin users and non-users in the placebo and Abiraterone arms**

|  | metformin (n= 104) | | | | No metformin (n=1081) | | | |
| --- | --- | --- | --- | --- | --- | --- | --- | --- |
|  | **AAP (n=73)** | | **Placebo (n=31)** | | **AAP (n=718)** | | **Placebo (n=363)** | |
| Toxicity | **Total (%)** | **Grade 3/4** | **Total** | **Grade 3/4** | **Total** | **Grade 3/4** | **Total** | **Grade 3/4** |
| Anemia | 22 (30.1%) | 10 (9.6%) | 11 (35.5%) | 2 (6.5%) | 189 (26.3%) | 57 (7.9%) | 101 (27.8%) | 30 (8.3%) |
| Thrombocytopenia | 1 (1.4%) | 1 (1%) | 1 (3.2%) | 0 (0%) | 30 (4.2%) | 10 (1.4%) | 14 (3.9%) | 2 (0.6%) |
| Neutropenia | 3 (4.1%) | 0 (0%) | 1 (3.2%) | 1 (3.2%) | 7 (1.0%) | 2 (0.3%) | 1 (0.3%) | 0 (0%) |
| Febrile neutropenia | 0 | 0 (0%) | 0 | 0 (0%) | 3 (0.4%) | 3 (0.4%) | 0 | 0 (0%) |
| Diarrhea | 22 (30.1%) | 2 (1.9%) | 4 (12.9%) | 0 (0%) | 147 (20.5%) | 8 (1.1%) | 55 (15.2%) | 5 (1.4%) |
| Fatigue | 34 (46.6%) | 6 (5.8%) | 20 (64.5%) | 7 (22.6%) | 352 (49.0%) | 73 (10.2%) | 155 (42.7%) | 35 (9.6%) |
| Asthenia | 16 (21.9%) | 1 (1%) | 7 (22.6%) | 1 (3.2%) | 118 (16.4%) | 26 (3.6%) | 48 (13.2%) | 7 (1.9%) |
| Back pain | 28 (38.4%) | 5 (4.8%) | 11 (35.5%) | 4 (12.9%) | 255 (35.5%) | 55 (7.7%) | 131 (36.1%) | 37 (10.2%) |
| Nausea | 25 (34.2%) | 4 (3.8%) | 10 (32.3%) | 0 (0%) | 248 (34.5%) | 16 (2.2%) | 123 (33.9%) | 11 (3%) |
| Vomiting | 21 (28.8%) | 2 (1.9%) | 9 (29.0%) | 0 (0%) | 188 (26.2%) | 20 (2.8%) | 93 (25.6%) | 13 (3.6%) |
| Hematuria | 8 (11.0%) | 3 (2.9%) | 4 (12.9%) | 1 (3.2%) | 72 (10.0%) | 10 (1.4%) | 30 (8.3%) | 8 (2.2%) |
| Abdominal pain | 10 (13.7%) | 1 (1%) | 4 (12.9%) | 0 (0%) | 104 (14.5%) | 19 (2.6%) | 43 (11.8%) | 8 (2.2%) |
| Pain in extremity | 12 (16.4%) | 2 (1.9%) | 6 (19.4%) | 1 (3.2%) | 160 (22.3%) | 26 (3.6%) | 79 (21.8%) | 21 (5.8%) |
| Dyspnea | 13 (17.8%) | 3 (2.9%) | 4 (12.9%) | 0 (0%) | 110 (15.3%) | 16 (2.2%) | 47 (12.9%) | 9 (2.5%) |
| Constipation | 26 (35.6%) | 2 (1.9%) | 10 (32.3%) | 0 (0%) | 211 (29.4%) | 9 (1.3%) | 116 (32.0%) | 5 (1.4%) |
| Pyrexia | 11 (15.1%) | 1 (1%) | 2 (6.5%) | 0 (0%) | 80 (11.1%) | 3 (0.4%) | 34 (9.4%) | 5 (1.4%) |
| Arthralgia | 30 (41.1%) | 7 (6.7%) | 8 (25.8%) | 2 (6.5%) | 218 (30.4%) | 39 (5.4%) | 87 (24.0%) | 15 (4.1%) |
| Urinary tract infection | 9 (12.3%) | 4 (3.8%) | 2 (6.5%) | 1 (3.2%) | 102 (14.2%) | 22 (3.1%) | 28 (7.7%) | 2 (0.6%) |
| Pain | 6 (8.2%) | 1 (1%) | 1 (3.2%) | 0 (0%) | 34 (4.7%) | 7 (1%) | 20 (5.5%) | 8 (2.2%) |
| Bone pain | 19 (26.0%) | 7 (6.7%) | 7 (22.6%) | 0 (0%) | 207 (28.8%) | 45 (6.3%) | 110 (30.3%) | 30 (8.3%) |
| Peripheral oedema | 20 (27.4%) | 1 (1%) | 9 (29.0%) | 0 (0%) | 209 (29.1%) | 13 (1.8%) | 66 (18.2%) | 3 (0.8%) |
| Hypokalemia | 17 (23.3%) | 6 (5.8%) | 6 (19.4%) | 1 (3.2%) | 136 (18.9%) | 34 (4.7%) | 30 (8.3%) | 2 (0.6%) |
| Cardiac disorder | 16 (21.9%) | 5 (4.8%) | 3 (9.7%) | 0 (0%) | 106 (14.8%) | 36 (5%) | 31 (8.5%) | 7 (1.9%) |
| Liver function test abnormalities | 0 | 0 (0%) | 0 | 0 (0%) | 0 | 0 (0%) | 1 (0.3%) | 1 (0.3%) |
| Hypertension | 6 (8.2%) | 1 (1%) | 4 (12.9%) | 1 (3.2%) | 76 (10.6%) | 12 (1.7%) | 23 (6.3%) | 0 (0%) |

**Supplemental Table 6: Toxicity rates in COU-AA-302 among metformin users and non-users in the placebo and Abiraterone arms**

|  | Metformin (n= 134) | | | | No metformin (n=948) | | | |
| --- | --- | --- | --- | --- | --- | --- | --- | --- |
|  | **AAP (n=66)** | | **Placebo-P (n=68)** | | **AAP (n=476)** | | **Placebo (n=472)** | |
| Toxicity | **Total (%)** | **Grade 3/4** | **Total** | **Grade 3/4** | **Total** | **Grade 3/4** | **Total** | **Grade 3/4** |
| Anemia | 17 (25.8%) | 4 (6.1%) | 11 (16.2%) | 2 (2.9%) | 46 (9.7%) | 11 (2.3%) | 42 (8.9%) | 8 (1.7%) |
| Thrombocytopenia | 0 | 0 (0%) | 1 (1.5%) | 0 (0%) | 11 (2.3%) | 2 (0.4%) | 7 (1.5%) | 4 (0.8%) |
| Neutropenia | 0 | 0 (0%) | 0 | 0 (0%) | 3 (0.6%) | 2 (0.4%) | 4 (0.8%) | 2 (0.4%) |
| Febrile neutropenia | 0 | 0 (0%) | 0 | 0 (0%) | 0 | 0 (0%) | 1 (0.2%) | 1 (0.2%) |
| Diarrhea | 20 (30.3%) | 1 (1.5%) | 22 (32.4%) | 0 (0%) | 117 (24.6%) | 7 (1.5%) | 76 (16.1%) | 5 (1.1%) |
| Fatigue | 37 (56.1%) | 3 (4.5%) | 30 (44.1%) | 2 (2.9%) | 207 (43.5%) | 13 (2.7%) | 170 (36.0%) | 10 (2.1%) |
| Asthenia | 7 (10.6%) | 0 (0%) | 7 (10.3%) | 0 (0%) | 39 (8.2%) | 1 (0.2%) | 40 (8.5%) | 6 (1.3%) |
| Back pain | 27 (40.9%) | 3 (4.5%) | 27 (39.7%) | 2 (2.9%) | 173 (36.3%) | 15 (3.2%) | 154 (32.6%) | 19 (4%) |
| Nausea | 15 (22.7%) | 0 (0%) | 18 (26.5%) | 0 (0%) | 129 (27.1%) | 6 (1.3%) | 109 (23.1%) | 1 (0.2%) |
| Vomiting | 11 (16.7%) | 1 (1.5%) | 7 (10.3%) | 0 (0%) | 76 (16.0%) | 3 (0.6%) | 54 (11.4%) | 0 (0%) |
| Hematuria | 7 (10.6%) | 2 (3%) | 3 (4.4%) | 1 (1.5%) | 55 (11.6%) | 5 (1.1%) | 30 (6.4%) | 2 (0.4%) |
| Abdominal pain | 7 (10.6%) | 0 (0%) | 9 (13.2%) | 2 (2.9%) | 45 (9.5%) | 5 (1.1%) | 40 (8.5%) | 7 (1.5%) |
| Pain in extremity | 14 (21.2%) | 1 (1.5%) | 19 (27.9%) | 1 (1.5%) | 93 (19.5%) | 3 (0.6%) | 70 (14.8%) | 4 (0.8%) |
| Dyspnea | 12 (18.2%) | 6 (9.1%) | 8 (11.8%) | 0 (0%) | 60 (12.6%) | 9 (1.9%) | 47 (10.0%) | 5 (1.1%) |
| Constipation | 19 (28.8%) | 0 (0%) | 21 (30.9%) | 0 (0%) | 125 (26.3%) | 2 (0.4%) | 91 (19.3%) | 3 (0.6%) |
| Pyrexia | 6 (9.1%) | 0 (0%) | 7 (10.3%) | 0 (0%) | 51 (10.7%) | 3 (0.6%) | 27 (5.7%) | 1 (0.2%) |
| Arthralgia | 25 (37.9%) | 1 (1.5%) | 22 (32.4%) | 2 (2.9%) | 148 (31.1%) | 10 (2.1%) | 110 (23.3%) | 8 (1.7%) |
| Urinary tract infection | 7 (10.6%) | 3 (4.5%) | 4 (5.9%) | 0 (0%) | 46 (9.7%) | 9 (1.9%) | 38 (8.1%) | 3 (0.6%) |
| Pain | 3 (4.5%) | 0 (0%) | 2 (2.9%) | 0 (0%) | 24 (5.0%) | 0 (0%) | 15 (3.2%) | 3 (0.6%) |
| Bone pain | 21 (31.8%) | 0 (0%) | 10 (14.7%) | 3 (4.4%) | 126 (26.5%) | 11 (2.3%) | 106 (22.5%) | 10 (2.1%) |
| Peripheral oedema | 19 (28.8%) | 0 (0%) | 22 (32.4%) | 2 (2.9%) | 130 (27.3%) | 3 (0.6%) | 97 (20.6%) | 3 (0.6%) |
| Hypokalemia | 10 (15.2%) | 3 (4.5%) | 10 (14.7%) | 1 (1.5%) | 91 (19.1%) | 11 (2.3%) | 59 (12.5%) | 9 (1.9%) |
| Cardiac disorder | 14 (21.2%) | 5 (7.6%) | 13 (19.1%) | 1 (1.5%) | 88 (18.5%) | 25 (5.3%) | 68 (14.4%) | 12 (2.5%) |
| Liver function test abnormalities | 0 | 0 (0%) | 0 | 0 (0%) | 4 (0.8%) | 0 (0%) | 0 | 0 (0%) |
| Hypertension | 14 (21.2%) | 4 (6.1%) | 4 (5.9%) | 1 (1.5%) | 115 (24.2%) | 21 (4.4%) | 70 (14.8%) | 16 (3.4%) |
